# Diagnosis in Disarray: A Rare Case of Leiomyoma of the Vulva

**DOI:** 10.7759/cureus.97172

**Published:** 2025-11-18

**Authors:** Varsha Raja Ayyanar, Vasudeo Ridhorkar, Ashay Suryawanshi

**Affiliations:** 1 Surgery, KIMS-Kingsway Hospitals, Nagpur, IND; 2 Urology, KIMS-Kingsway Hospitals, Nagpur, IND

**Keywords:** bartholin cyst, benign, labia minora, smooth muscle tumour, vulval leiomyoma

## Abstract

Extrauterine leiomyomas are more uncommon and difficult to diagnose. These smooth muscle cell-based tumours, which are histologically benign, often develop in the genitourinary tract (in the vulva, ovaries, urethra, and urinary bladder); however, they can develop in almost any anatomic region. As reported in the literature, vulval leiomyomas are rare and commonly misdiagnosed as Bartholin cysts prior to surgery. These painless, isolated, well-circumscribed smooth muscle tumours can afflict females of any age group. This report describes the case of a 25-year-old female who visited the hospital with complaints of a right labial swelling that had been there for five months, and a provisional diagnosis of a Bartholin cyst was made. The patient underwent an elective excision under short general anaesthesia and local infiltration. The histopathological report revealed a benign vulval leiomyoma as the final diagnosis.

## Introduction

Vulval leiomyomas are rare and commonly misdiagnosed as Bartholin cysts before surgery. In the existing literature, fewer than 160 instances have been documented [1]. Most vulvar leiomyomas are painless, isolated, and well-defined. They can affect females of any age, although the majority are between the ages of 30 and 60. According to histology, the smooth muscle found in erectile tissue, the walls of blood vessels, and the round ligament give rise to vulvar leiomyomas. Spindle-shaped cells are characteristic of typical vulvar leiomyomas; however, epithelioid-type tumours and other histological kinds have also been identified. We report a rare case of leiomyoma in the right upper part of the labia minora in a 25-year-old female, in which the histopathological examination following its elective excision showed the structure of a well-circumscribed spindle cell tumour and other microscopic features suggestive of vulval leiomyoma.

## Case presentation

History

A 25-year-old woman who had had a right labial lump for five months arrived at the hospital. Initially, the lump was smaller in size and asymptomatic. Later, the mass gradually increased in size and caused discomfort. Her only concern was the deviation in the stream of urine towards the left. There was no history of pain over the swelling, discharge, fever, or weight loss. The patient is nulliparous and has been married for a year. She is not known to have any comorbidities. Her menstrual cycle was regular, and she had not undergone any previous surgeries.

Clinical findings

**Figure 1:**
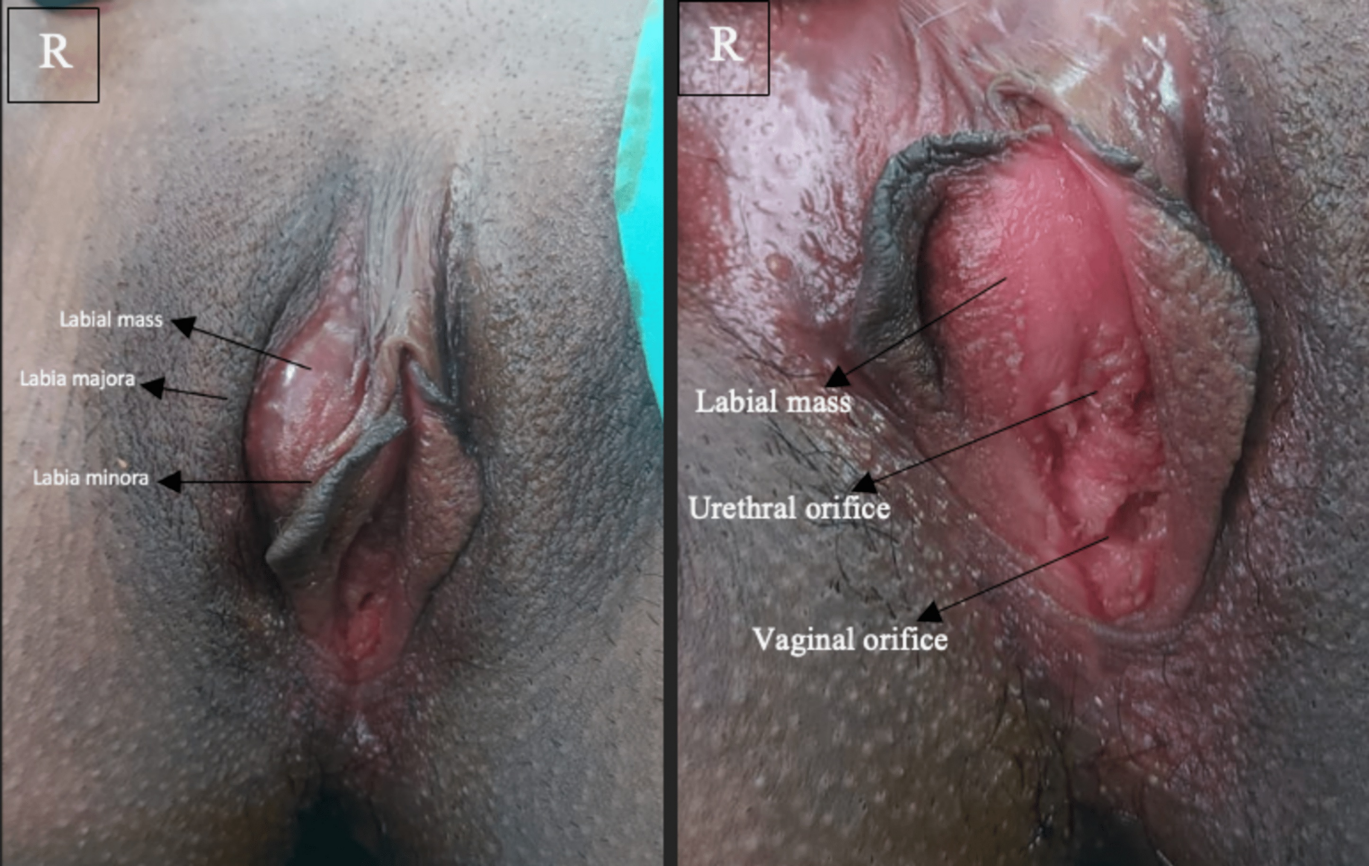
Gross examination findings

Except for a soft oval mass measuring 3 x 2 cm in the right vulval area, the general examination revealed nothing unusual. The lump was precisely lateral to the urethral orifice on the right labia minora (Figure 1). In compliance with ethical standards, the patient provided informed consent for the use of their pre-operative and intra-operative photographs in this educational material.

Surgical procedure

**Figure 2:**
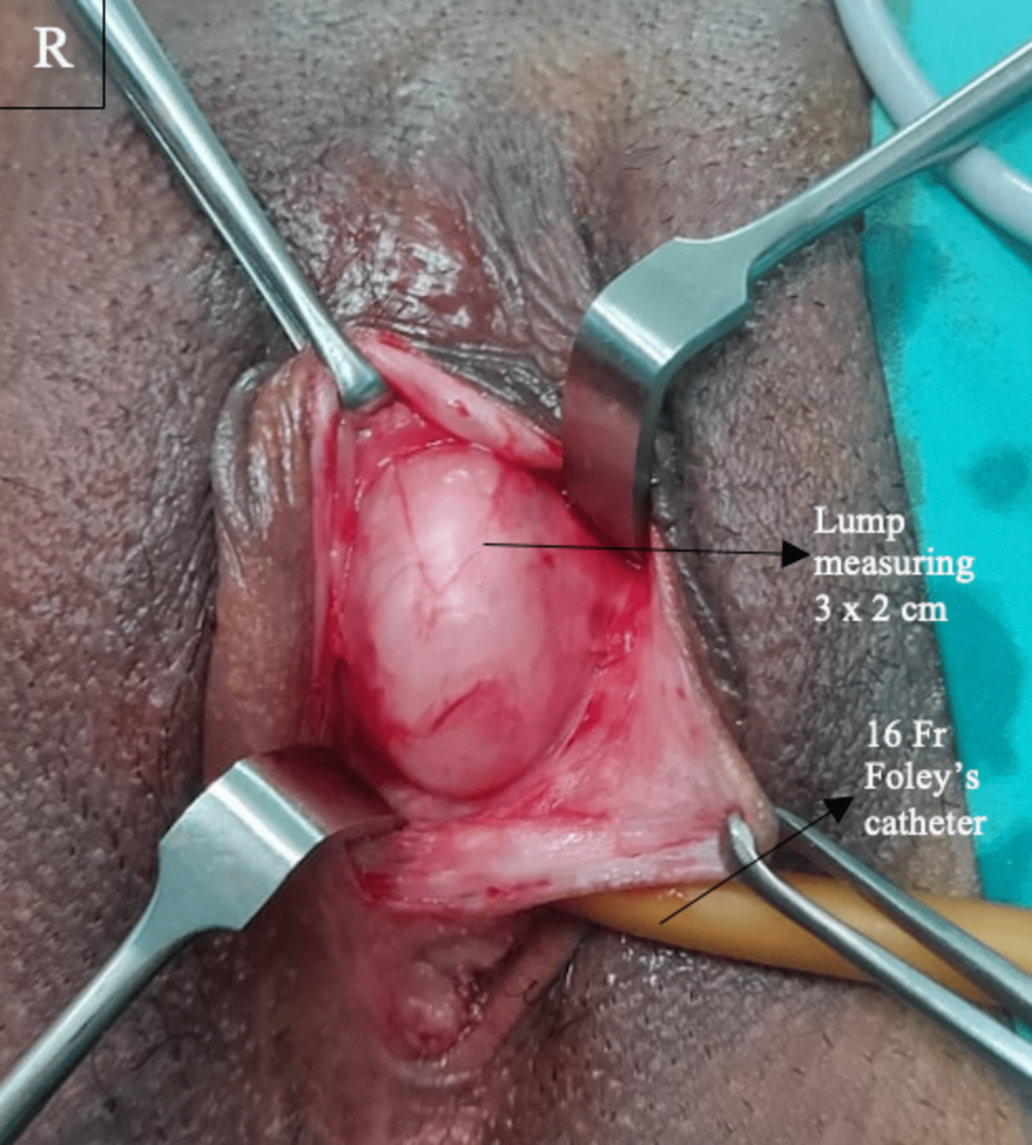
Intraoperative image of the lump

The mass was diagnosed initially as a Bartholin cyst. She sought counselling for surgical excision of the cyst and consented to the same. Under short general anaesthesia and local infiltration, an elective excision of the lump was carried out in the lithotomy position. The bladder was emptied by inserting a 16Fr Foley's catheter. A vertical incision was taken in the mucosal region of the labia minora, and a 3 x 2 cm soft, fleshy, and well-defined lump was identified (Figure 2). The mass had a definite capsule, and it was dissected free by sharp dissection. It was delivered intact with the capsule. The consistency on palpation appeared solid and fibrous, and the mass was sent to the histopathology lab for analysis. Following surgery, the patient had a satisfactory recovery. She was followed up post surgery with no complications.

Histopathology

**Figure 3:**
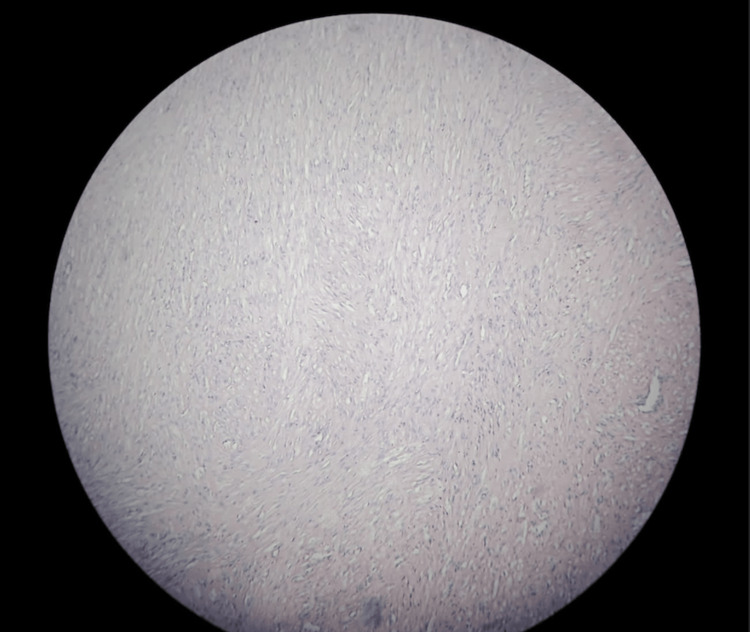
Benign spindle cell tumour arranged in interlacing fascicles These cells have indistinct cell membrane and moderate eosinophilic cytoplasm. No mitotic figures or necrosis seen.

On histopathological examination, the gross specimen consisted of a single labial soft tissue mass measuring 3.2 × 2.5 × 1 cm. The cut section of the specimen revealed a fleshy, solid cut surface that was white in colour and had a whorl appearance. No cyst was seen. Microscopy revealed a benign spindle cell tumour arranged in fascicles. The cells had an indistinct cell membrane and moderately eosinophilic cytoplasm. Areas of hyalinization are seen. No mitotic features or necrosis were seen (Figure 3). The final diagnosis was benign vulvar leiomyoma.

## Discussion

Leiomyomas are benign tumours that can develop from smooth muscle cells in any part of the body. Fewer than 160 cases of vulvar leiomyoma have been documented in the English literature to date; hence, it is an uncommon tumour [1-3]. It has several histological origins, including smooth muscle cells, spindle cells, and epithelioid cancer cells with eosinophilic cytoplasm. Vulvar leiomyomas show positive immunohistochemical staining for oestrogen, progesterone, or, occasionally, both oestrogen and progesterone receptors. Consequently, it may be advantageous to use receptor modulators in combination with surgery.

This case report demonstrates the value of an extensive history and thorough investigation. In sexually active women, Bartholin's cyst is frequently observed. It usually presents with a relatively painless swelling, and if the cystic fluid gets infected, it is called a Bartholin’s abscess. Any patient who was initially determined to have a Bartholin’s cyst or abscess has to be re-evaluated once the swelling goes down. On examining a Bartholin cyst, it has cystic consistency on palpation, and the labia minora are everted, whereas one can find an intact hymen, solid consistency, and an inverted labia minora that are suggestive of a leiomyoma [4]. In this instance, the patient's labia minora were everted despite the patient having a hard bulge, which caused perplexity. Although uncommon, the vulva can occasionally host smooth muscle tumours. Surgical excision of the tumour is the treatment of choice for these lesions.

It might be difficult to distinguish between benign and malignant tumours in cases of vulvar leiomyoma. Four characteristics were described by Nielsen et al. as a criterion to differentiate between the two lesions: a widest dimension of more than 5 cm, infiltrative margins, more than five mitotic figures per 10 hpf, and intermediate to severe cytologic atypia [5].

If three or all of the criteria are present, the neoplasm is thought to be a sarcoma. Only two of the criteria are met by benign but atypical leiomyomas, and simple benign leiomyomas show one or none of the qualities. According to their opinion, our case lacked all the aforementioned characteristics, which raised the possibility that it was a benign leiomyoma. Trans-perineal ultrasonography aids in the identification of vulval leiomyoma, whereas MRI aids in the distinction between benign and malignant forms in situations when the diagnosis is uncertain.

## Conclusions

Extrauterine leiomyomas are uncommon and more difficult to diagnose. Apart from the unusual sites of its origin mentioned above, it can also be rarely seen in the sino-nasal cavities, orbits, kidneys, and skin. Bartholin's cysts, fibromas, lymphangiomas, soft-tissue sarcomas, and neurogenic tumours are among the differential diagnoses. Conservative surgery is used to treat labial leiomyomas with simple excision and biopsy. Long-term follow-up is recommended after surgery.
